# Transfusion protocol in trauma

**DOI:** 10.4103/0974-2700.76844

**Published:** 2011

**Authors:** Paramjit Kaur, Sabita Basu, Gagandeep Kaur, Ravneet Kaur

**Affiliations:** Blood Bank, GGS Medical College and Hospital, Faridkot, India; 1 Department of Transfusion Medicine, Government Medical College and Hospital, Sector 32, Chandigarh, India

**Keywords:** Blood transfusion, massive transfusion, resuscitation, trauma

## Abstract

Blood and blood components are considered drugs because they are used in the treatment of diseases. As with any drug, adverse effects may occur, necessitating careful consideration of therapy. Like any other therapeutic decision, the need for transfusion should be considered on the basis of risks and benefits and alternative treatments available to avoid over- and under-transfusion. This review is focused on the blood transfusion protocol in trauma patients with hemorrhagic shock. Besides, issues related to emergency and massive transfusion have also been elaborated. We conducted a comprehensive MEDLINE search and reviewed the relevant literature, with particular reference to emergency medical care in trauma.

## INTRODUCTION

Blood transfusion services are a vital and integral part of modern health care. Reducing patient morbidity and mortality is a key objective of any surgical or anesthetic practice. The decision to transfuse a patient can often be a difficult judgment. Resuscitation of a patient with trauma and hemorrhagic shock is the responsibility of the emergency physicians, surgeons, and anesthesiologists. Doctors in these specialties often have to manage life-threatening hemorrhage in addition to the underlying pathology and in such situations are forced to take complex decisions. A good understanding of the physiology of shock and the implications of treatment strategies is essential to achieve a favorable outcome in such patients. In some surgical procedures significant blood loss can be anticipated, and there is always the potential for unexpected blood loss during any type of surgery.

Transfusion support is vital for a patient of trauma with hemorrhagic shock. The transfusion of the right component to the right patient in the right quantity and at the right time has been the main focus of attention for critical care doctors. Although blood is the ideal resuscitation agent from a physiological perspective, there are limitations to its use. The potential for disease transmission with blood and blood products is a major concern. In spite of stringent screening of the blood supply with modern sensitive techniques, there is no ‘zero risk’ transfusion. Besides, other concerns such as availability of components, cost, shelf life, and even religious prohibitions against transfusion of blood products limit the use of such components.

## PATHOPHYSIOLOGY OF HEMORRHAGIC SHOCK

Significant hemorrhage can overcome the normal compensatory mechanisms, compromise oxygenation and tissue perfusion, and lead to hemorrhagic shock. Besides trauma victims, surgical and obstetric cases constitute the majority of such patients. Acute hemorrhage triggers a series of physiologic responses involving multiple organ systems, including the cardiovascular, respiratory, renal, hematologic, and neuroendocrine systems. Pain, cognitive perception of an injury, and reduction of cardiac preload results in the fight or flight response. A fall in the blood pressure is one of the very first responses. This cannot be attributed only to the initial reduction in intravascular volume.[1] There is a sudden reduction in the peripheral vascular resistance, which causes a fall in the blood pressure. The vagal tone decreases due to stimulation of baroreceptors and this leads to an increase in heart rate and cardiac output. Sympathetic outflow and catecholamine release causes constriction of nonessential vascular beds and increases cardiac inotropy.[2] Thus, a functional reserve is provided to compensate for acute blood loss.

When there is significant blood loss, there is stimulation of the endocrine system with release of antidiuretic hormone. This helps in conservation of sodium and water. With a drop in the blood pressure, there is activation of the juxtaglomerular apparatus, resulting in secretion of rennin. This forms angiotensin II, which is responsible for vasoconstriction and stimulation of the adrenal cortex to release aldosterone. Aldosterone helps in conservation of sodium and water and also stimulates hydrogen ion secretion, with reduction in acidosis.

Other effects of acute hemorrhage are local activation of the coagulation system and adherence of platelets at the site of the damage in the vessels. This, in association with regional reduction in the blood flow by systemic hypotension and local vasoconstriction, can lead to the formation of a stable clot at the site of bleeding.

All these compensatory mechanisms maintain critical organ perfusion even in severe hemorrhage. But persistent hemorrhage will overwhelm the capacity of systemic compensatory mechanisms and lead to a downward spiral in the pathophysiology.[3] A decrease in oxygen delivery at the cellular level leads to anaerobic metabolism, fluid uptake from extracellular space, cellular hibernation, and cell death.[4] Cellular ischemia causes release of inflammatory mediators and metabolic byproducts into the circulation. Lack of sufficient energy reserves to maintain a vasoconstricted state leads to the irreversible stage, with development of the lethal triad of acidosis, coagulopathy, and hypothermia and this heralds the failure of resuscitation.[5]

If the hemorrhage is controlled, the outcome of an episode of shock is influenced by the rate and precision of fluid resuscitation, the cellular response of individual organ systems, and the balance of inflammatory mediators over the subsequent hours or days. The patient may survive acutely but die days to weeks later of multiple organ failure triggered by the initial episode of shock.

## FLUID RESUSCITATION

Fluid resuscitation and achievement of hemostasis is the priority in a patient with hemorrhagic shock. Although reduction of the hematocrit is a marker of hemorrhage, it may be misleading in case of rapid hemorrhage. Rapid administration of fluid and blood products to support systemic perfusion can lead to excessive hemorrhage or rebleeding from previously hemostatic areas[6] due to increased myocardial contractility, elevated blood pressure, and reversal of vasoconstriction in injured tissues.[7] Fluid resuscitation may be detrimental if it is started before bleeding is controlled in patients with trauma. In hypotensive patients with penetrating torso injuries, if aggressive fluid resuscitation is delayed until operative intervention the outcome is better.[8] In a randomized controlled study the authors found that vigorous infusion of normal saline after massive splenic injury resulted in a significant increase in intra-abdominal bleeding and shortened survival time. The hemodynamic response to crystalloid infusion in blunt abdominal trauma depends on the severity of injury and the rate of blood loss.[9]

The choice of the resuscitation fluid is equally important; there must be maintenance of adequate intravascular volume, oxygen-carrying capacity, clotting function, and electrolyte balance and, at the same time, minimization of extravascular edema and immune system activation. Although crystalloid administration in the form of normal saline or Ringer’s lactate are the immediate means of resuscitation at most centers, careful titration of the quantity infused is required to avoid the negative consequences of over-infusion. Only about 30% of infused crystalloid remains intravascular and therefore the volume required to be infused is about three times that of the lost blood.[10]

Colloids have osmotic activity and therefore the potential for expanding the intravascular volume. It has been proposed that the osmotic effect also helps in maintenance of microvascular perfusion.[11] However, the choice of fluid for initial resuscitation still depends upon logistic factors such as expense and ease of use. In one study, the effect of crystalloid and colloid infusion was studied following massive splenic injury. The authors concluded that the hemodynamic response to crystalloid or colloid infusion in blunt abdominal trauma is primarily dependent on the severity of injury and the rate of fluid resuscitation.[12] However, various randomized control trials comparing crystalloid *vs* colloid for resuscitation in critically ill patients (with trauma, burns, or following surgery) found no evidence that resuscitation with colloids reduces the risk of death compared to resuscitation with crystalloids. As colloids are not associated with an improvement in survival and, besides, are more expensive than crystalloids, their continued use in these patients is not advocated.[13] Randomized and quasi-randomized trials comparing colloid solutions in critically ill and surgical patients thought to need volume replacement also did not conclude any particular colloid solution to be safe when comparing outcomes like death, amount of whole blood transfused, and incidence of adverse reactions.[14]

Voluven^®^ (hydroxyethyl starch [HES] 130/0.4), a new-generation HES product with low molecular weight, has been used for the treatment of traumatic and hemorrhagic shock. It is believed that early infusion of Voluven^®^ is beneficial for maintenance of hemodynamic stability and to balance cerebral oxygen supply and consumption during the resuscitation phase of acute hemorrhagic shock.[15] New therapeutic strategies, like immunomodulation, cardiovascular maintenance and small-volume resuscitation have either been introduced or are in the process of being transferred from bench to bedside.[16]

## BLOOD TRANSFUSION

The decision to transfuse should not only be based on transfusion triggers but should be coupled with adequate knowledge of the clinical symptoms, rate and extent of ongoing blood loss, cardiac function, and the need for operative intervention. The end goal of transfusion is to restore volume and oxygen-carrying capacity. The type of component to be transfused depends on assessment of the clinical status of the patient.

### Whole blood

With the advent of blood component therapy, the use of whole blood as a resuscitation fluid has become obsolete. Whole blood is deficient in clotting factors and has high levels of potassium, ammonia, and hydrogen ions. Although it provides volume expansion along with increased oxygen-carrying capacity, there can be volume overload before the needed components are replenished.

### Packed red blood cells

Since the central pathophysiology of hemorrhagic shock is failure of oxygen delivery, timely administration of red blood cells is the most important component of resuscitation. Blood loss greater than 25% to 30% usually requires transfusion of packed red blood cells in addition to crystalloids.[17] Ensuring a ready supply of type ‘O’ blood that can be immediately delivered to the bedside can be life saving in the rapidly exsanguinating patient.[18]

### Fresh frozen plasma

Fresh frozen plasma (FFP) is utilized for its clotting factor content in trauma resuscitation. In the presence of massive hemorrhage or coagulopathy, 1 unit of FFP is given for every 4 or 5 units of red cells administered.[19] Administration of FFP should be guided by serial measurement of clotting times, fibrinogen levels, prothrombin time (PT), and activated partial thromboplastin time (APTT). FFP is not indicated just for volume expansion in trauma cases. However, a more proactive approach is beneficial in rapid bleeding to prevent the development of a coagulopathy. Hemotherapy decisions should be inclined towards reducing the magnitude of a problem before it worsens to the point of no return. Adhering to set formulae for plasma or platelet transfusions may aggravate the patient’s bleeding. Besides assessment of patient factors such as reduced body temperature and presence of acidosis, both of which decrease the *in vivo* activity of the coagulation system,[20] other methods such as use of thromboelastography analysis of the blood’s ability to clot can assist in recognition of a problem.[21] In addition, the precise prediction of the dosage of plasma needed to correct the coagulopathy is another challenging task. Change in the prothrombin time is exponentially related to the factor activity and is faster when the patient’s procoagulant activity is very different from normal.[22] The timing of plasma transfusion is also important. If correction is required before a hemostatic challenge such as a major surgery, it should be given shortly before the procedure for maximum benefit.

### Platelets

The decision to transfuse platelets should be based on the etiology of the thrombocytopenia, the presence or absence of active bleeding, and the need for surgical intervention. The trigger for starting platelet transfusion is also undecided. Generally, when the platelet count is below 10 000/µl, platelets are transfused prophylactically to prevent spontaneous hemorrhage. If the patient is bleeding or if invasive intervention is planned and the count is between 10 000/µl and 50 000/µl, then platelet transfusion should be given. In such patients, the count should be maintained above 50 000/µl. Platelet therapy should be guided by monitoring of the post-transfusion platelet count. Patients with fever, infections, disseminated intravascular coagulation, excessive bleeding, and splenomegaly may not show the expected increase in platelet count following transfusion.[17]

### Hemoglobin-based oxygen carriers

Hemoglobin-based oxygen carriers (HBOCs) represent an attempt to create a resuscitative fluid with the oxygen-carrying capacity of red blood cells but without the need for cross-matching or the potential for viral transmission. These solutions are either human or bovine in origin and consist of hemoglobin dimers or tetramers. They have shorter half-lives than red blood cells (hours to days).[23,24] Studies have shown promising results, demonstrating that HBOCs are capable of replacing all or part of the transfusion requirement in surgery.[25,26] One potential disadvantage is elevation of systolic blood pressure by vasoconstriction. Ongoing research into the use of HBOCs in lieu of blood transfusion in trauma patients may prove their effectiveness in the near future.

### Recombinant erythropoietin

Hemorrhagic shock leads to suppression of erythrocyte production due to the effect of cytokines released as a part of the systemic inflammatory response.[27] High levels of erythropoietin achieved by recombinant erythropoietin administration have established a robust erythrocyte response in the seriously injured patient.[28] The early provision of recombinant erythropoietin to trauma patients may reduce the transfusion requirement significantly. In a prospective randomized double-blind placebo-controlled multicenter trial, it was found that weekly administration of recombinant human erythropoietin reduces allogeneic red cell transfusions in critically ill patients. There was an increase in the hemoglobin and hematocrit values in the study group, which included both medical and surgical patients.[29] Other authors have also reported similar observations with recombinant erythropoietin.[30] The EPO Critical Care Trials Group observed reduced mortality in trauma patients treated with erythropoietin-alfa. However, the treatment is associated with significant increase in the number of thrombotic events.[31] The dilemma for the trauma surgeon faced with life-threatening blood loss in a Jehovah’s Witness who refuses blood transfusions due to religious prohibitions can be resolved with the use of recombinant human erythropoietin in combination with other blood conservation techniques.[32,33]

### Cryoprecipitate

Cryoprecipitate is obtained by slowly thawing FFP. It is rich in factor VIII, von Willebrand factor, and fibrinogen.[34] If FFP is used to supplement massive transfusion, cryoprecipitate may not be required unless fibrinogen level falls to below 100 mg/dl.[35] Although cryoprecipitate can rapidly increase the concentration of fibrinogen and Von Willebrand factor, the advantages of cryoprecipitate in high concentration in the massively bleeding trauma patient are not proven.[36]

### Factor VIIa

Recombinant factor VIIa is being used in the treatment of bleeding in trauma and non-trauma patients when all other measures fail. It can stop blood loss, reduce blood requirement, and improve clotting parameters.[37] A randomized study in trauma patients recently demonstrated that adjunctive therapy with recombinant factor VIIa controls bleeding, resulting in a significant reduction in red blood cell transfusion need in trauma.[38] The authors further evaluated the pharmacokinetic properties of the factor in trauma patients. They suggested that dosing be adjusted according to the clinically estimated blood loss and the rate of bleeding.[39] Further studies are required before recombinant factor VIIa can be recommended for trauma patients.

### Desmopressin acetate

Desmopressin acetate (DDAVP) initiates the release of von Willebrand factor and can increase von Willebrand factor and factor VIII levels in normal subjects 3–5 fold.[40] It can be utilized in conjunction with other components in patients with associated coagulopathy.

## SPECIAL CIRCUMSTANCES

### Emergency transfusion

Urgent need for transfusion in a patient requiring immediate surgical intervention may preclude the performance of usual testing protocol. Adequate pretransfusion samples should be collected before infusion of any donor blood so that compatibility testing, antibody screening and, if necessary, identification studies can be performed subsequently. If blood must be issued in an emergency and there is no time for cross-matching, group-specific blood can be issued. In extreme emergencies, when there is no time to obtain and test a sample, group ‘O’ Rh-negative packed red cells can be released. In such a situation the clinician must sign a release authorizing and accepting responsibility for the use of incompletely tested products as a life-saving measure.

### Massive transfusion

Massive transfusion is defined as transfusion approximating or exceeding the patient’s blood volume, or transfusion of more than 10 units of blood within 24 h.[41] Replacement of more than 50% of circulating blood volume in less than 3 h or transfusion at the rate of more than 150 ml/min is also considered as massive transfusion.[42] The need for massive (or large volume) transfusions generally arise as a result of acute hemorrhage in surgical and trauma patients. A blood loss of less then 20% of the total blood volume is generally well tolerated, while a loss of 20% to 40% will cause change in the vital signs, with evidence of impaired tissue perfusion. However, loss of more than 40% of blood volume may lead to frank hemorrhagic shock and progress to circulatory system failure and cardiac arrest if not corrected.[43]

The total volume and type of fluid infused during initial resuscitation has a strong influence on the outcome. Priority should be given to maintenance of intravascular volume and adequate oxygen-carrying capacity. Other parameters such as clotting factors and serum electrolyte levels should also be monitored.[44,45] Administration of blood and blood products is a central feature of the treatment regimen for patients with hemorrhagic shock. Whereas all transfusions have potential adverse reactions, the transfusion of massive amounts of stored blood is associated with unique consequences, such as shift to the left in the oxygen dissociation curve, acid-base imbalance, hypothermia, hypocalcaemia, dilutional coagulopathy, and respiratory distress. The American College of Surgeons and the American Association of Blood Banks both recommend that the transfusion of blood and blood components be guided by laboratory tests such as PT, PTT, platelet count, and fibrinogen levels. Only a minimum level of coagulation factors are required for normal formation of fibrin and hemostasis and normal plasma can therefore be said to contain coagulation factors in excess, a reserve that usually allows patients to tolerate replacement of one or more blood volumes of red cells and crystalloid without needing FFP. Guidelines typically state that the threshold for therapeutic or prophylactic FFP is a PT 1.5 times the upper limit of normal or the mid-point of the normal range and a PTT 1.5 times the upper limit of normal in an appropriate clinical setting.[46,47] In the event of microvascular bleeding, empiric therapy with platelets and/or plasma may be initiated immediately after specimens are obtained. In consumptive coagulopathy, a platelet count of less than 50 000/µl and a fibrinogen level less than 100 mg/dl are better predictors of hemorrhage than PT and aPPT.[48] Supplementation with FFP should be considered after one volume is lost and definitely started before blood loss equals 150% of total volume. At this stage, 4–5 units of FFP should be infused and, subsequently, 4 units FFP for every 6 units of red cells.[47] Some authors suggest that massive transfusion protocols should utilize a 1:1 ratio of plasma to red blood cells for all patients who are hypocoagulable following traumatic injuries.[49] In a large multicenter retrospective analysis, the authors concluded that early administration of high ratios of FFP and platelets improves survival and decreases overall red blood cell need in massively transfused patients. There was a significant difference in mortality during the first 6 h after admission. Thus early administration of FFP and platelets is critical in trauma with massive bleeding.[50] The current massive transfusion protocols are changing worldwide. These being a key element of damage control, early and aggressive transfusion intervention and resuscitation with blood components is the novel approach to major trauma that addresses the lethal triad of acidosis, coagulopathy, and hypothermia. Although the ideal amounts of plasma, platelet, cryoprecipitate, and other coagulation factors in relationship to the red blood cell transfusion volume are not known, current data support a target ratio of plasma : red blood cell : platelet transfusions of 1:1:1.[51]

## CONCLUSION

Initial resuscitation of a patient with hemorrhagic shock should be based on identification and correction of the source of bleeding and fluid administration to stop, and then reverse, the pathophysiology of shock. The rate of administration of resuscitation fluids should be adequate to support tissue perfusion. Early use of blood component therapy can help to preserve oxygen delivery and coagulation parameters. However, blood transfusion carries the added risk of transfusion reactions, infections, and several metabolic complications in massive transfusion. Hence, the indiscriminate or prophylactic use of blood products is not warranted.
